# Speculation on the Mechanism of Parkinson’s Disease Induced by Risk Residual Pesticides in Fresh Jujube and Hawthorn Through Network Toxicology and Molecular Docking Analysis

**DOI:** 10.3390/foods14193324

**Published:** 2025-09-25

**Authors:** Yecan Pan, Wenkui Liu, Wenxin Shi, Ying Lv, Chen Yang, Yanjie Wang, Chao Ding, Bianqing Hao

**Affiliations:** Shanxi Center for Testing of Functional Agro-Products, Shanxi Agricultural University, Taiyuan 030031, China; panyecan.love@163.com (Y.P.); 16635570447@163.com (Y.L.); 15035168995@163.com (C.Y.); 18335579193@163.com (Y.W.)

**Keywords:** pesticide residues, network toxicology, molecular docking, Parkinson’s disease (PD), fresh jujube, hawthorn

## Abstract

Parkinson’s disease (PD) is a neurodegenerative disease that is closely related to genetic and environmental factors, among which pesticide exposure is considered an important risk factor. Fresh jujube and hawthorn, as widely consumed fruits, may contain pesticide residues, but the potential effects of long-term low-dose intake on PD are not yet clear. This study combines network toxicology and molecular docking technology to elucidate the molecular mechanism of PD induced by residual pesticides in fresh jujube and hawthorn. Firstly, common risk pesticides (such as organophosphates and pyrethroids) in fresh jujube and hawthorn were screened through the database. Subsequently, a “pesticide target—PD” interactive network was constructed using network toxicology to predict key targets and related pathways. Finally, molecular docking technology was used to verify the binding ability of pesticide molecules to PD-related proteins. The results indicate that some pesticides (such as chlorpyrifos and cypermethrin) may increase the risk of PD by affecting lipid metabolism and oxidative stress response. This study provides a new approach for assessing the neurotoxicity of pesticide residues and suggests the need to pay attention to the potential impact of dietary pesticide exposure on PD, providing a scientific basis for food safety regulation and PD prevention strategies.

## 1. Introduction

Parkinson’s disease (PD) is a prevalent neurodegenerative disorder characterized by the progressive loss of dopaminergic neurons in the substantia nigra of the midbrain, along with the abnormal aggregation of alpha-synuclein in Lewy bodies [1,2,3]. The pathogenesis of PD is multifaceted, involving various factors such as genetic susceptibility [4,5,6], exposure to environmental toxins [7,8], mitochondrial dysfunction [9,10], oxidative stress [11,12], gut microbiome [13,14,15], and neuroinflammation [16,17,18]. While approximately 10% of PD cases are linked to genetic mutations, the majority are classified as sporadic, with environmental factors playing a crucial role in their onset [19,20,21].

Pesticides, widely utilized in agriculture, have been implicated in an increased risk of PD through various epidemiological and experimental studies [22,23]. Organophosphorus pesticides (e.g., phorate and fenitrothion), organochlorine pesticides (e.g., dieldrin and lindane), and pyrethroid pesticides (e.g., deltamethrin and fenvalerate) can traverse the blood–brain barrier, disrupt neurotransmitter metabolism, induce oxidative stress, and promote alpha-synuclein aggregation, thereby accelerating the pathological progression of PD [24,25,26,27]. A research team from the School of Public Health at Zhejiang University employed paraquat to induce PD-like changes, such as lipid metabolism disorders and inflammation, in a murine model. Their findings confirm that paraquat triggers the pathogenesis of PD by modulating inflammatory factors and inflammatory lipid compounds [28]. This study emphasizes the critical importance of increasing surveillance in order to reduce pesticide exposure and safeguard public health. Nevertheless, the majority of existing research has concentrated on occupational exposure to high-dose pesticides, leaving a gap in studies regarding the neurotoxicity of pesticide residues resulting from long-term low-dose dietary intake.

Fresh jujube (*Ziziphus jujuba*) and hawthorn (*Crataegus pinnatifida*) are widely consumed fruits in China, recognized for their significant medicinal and nutritional value. These fruits are abundant in active compounds, including vitamins, polyphenols, and flavonoids, which exhibit antioxidant, anti-inflammatory, and neuroprotective properties [29,30,31,32,33]. However, the prevalent use of pesticides in agricultural practices to combat pests and diseases can lead to the accumulation of pesticide residues in fresh jujubes and hawthorns. While the residual levels typically comply with national food safety standards, prolonged consumption of low doses of pesticides may elevate the risk of PD through cumulative or synergistic effects with other environmental toxins. Therefore, conducting a systematic evaluation of the neurotoxicity of pesticide residues in fresh jujube and hawthorn, as well as elucidating their potential mechanisms for inducing PD, is crucial for enhancing food safety regulations and preventing PD.

**Figure 1:**
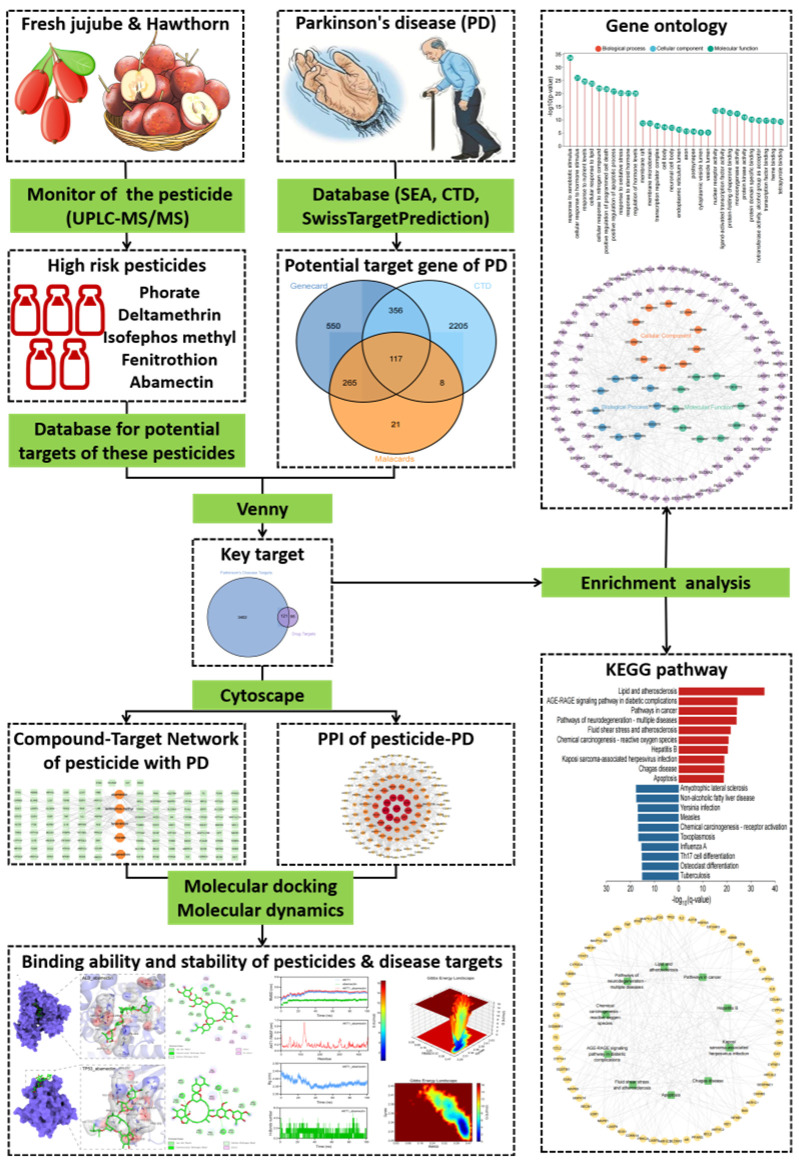
Flow chart showing the experimental design.

In recent years, computational toxicology methods, such as network toxicology and molecular docking techniques, have emerged as efficient and cost-effective strategies for investigating the relationship between environmental toxins and diseases [34,35]. Network toxicology systematically predicts the potential mechanisms of toxic substances by integrating the interaction networks of compounds, targets, pathways, and diseases [36]. Additionally, molecular docking technology simulates the binding modes between toxin molecules and key target proteins at the atomic level, thereby validating their interaction capabilities [37,38]. This study aims to integrate two methodologies. First, common residual pesticides and their potential neurotoxic components in fresh jujube and hawthorn will be screened. Subsequently, a pesticide target—PD network will be constructed to identify key pathways and core targets. Finally, molecular docking will be employed to analyze the binding affinity of pesticide molecules with PD-related proteins. This research seeks to provide novel insights for the neurotoxicity assessment of pesticide residues, to supplement the scientific basis for the environmental etiology of PD, and to offer theoretical support for optimizing food safety standards and early prevention strategies for PD. The complete research process is depicted in Figure 1.

## 2. Materials and Methods

### 2.1. Screening of Potentially Hazardous Pesticide Residues in Fresh Jujube and Hawthorn

In different regions of Shanxi Province, various varieties of hawthorn and fresh jujubes were collected at different times for the screening of pesticide residues with potential risks. A sample of 10.00 g, after being ground and homogenized, was placed in a 50 mL centrifuge tube. Then, 10 mL of acetonitrile (Merck Co., Ltd., Rahway, NJ, USA, hypergrade for LC-MS) containing 2% acetic acid (Merck Co., Ltd., Rahway, NJ, USA, hypergrade for LC-MS) was added, and the mixture was vortexed at a speed of 2500 rpm for 10 min to extract. Subsequently, a QuEChERS Extraction Packets (4 g of anhydrous magnesium sulfate, 1 g of sodium chloride, 1 g of sodium citrate, and 0.5 g of disodium hydrogen citrate, SHIMADZU Co., Ltd., Kyoto, Japan) was added. After quickly shaking to mix, the sample was vortexed at 2500 rpm for 5 min and then centrifuged at 8000 rpm for 3 min. After purification with 1.5 mL of supernatant, the mixture was subjected to vortex dispersion solid-phase extraction at 2500 rpm for 5 min, followed by centrifugation at 5000 rpm for 2 min. The supernatant was then filtered through a 0.22 µm organic filter membrane and detected by UPLC-MS/MS.

The liquid chromatography was carried out using a binary LC pump (ExionLC, AB Sciex, Framingham, MA, USA) equipped with an autosampler (ExionLC AD, AB Sciex, Framingham, MA, USA). Analytes were chromatographed on an ACQUITY UPLC^®^ BEH C-18 column (100 mm × 2.1 mm i.d, 1.7 mm particle size) (Waters, Milford, MA, USA). The analysis was carried out using a solvent gradient. The mobile phase A is a 4 mmol/L ammonium acetate aqueous solution (containing 0.1% formic acid); B is methanol. The initial composition of the mobile phase ranged from 5% B to 20% B in 1 min, then from 1.00 to 1.10 min at 40.0% B, 1.10–3.00 min to 60.0% B, 3.00–3.10 min to 80.0% B, 3.10–5.00 min to 95.0% B, 5.00–7.00 min hold at 95.0% B, 7.00–9.00 min return to 5.0% B;, and until 10.00 min hold at 5.0% B. The return to 5.0% B occurred in 2 min and this composition was maintained for 1 min before the next injection. The total run time was 10 min and the injection volume was 2 μL. The flow rate of the mobile phase was 0.3 mL min^−1^. The column effluent was introduced into the ESI source of the MS/MS system (Triple Quad 4500, AB Sciex, Framingham, MA, USA). The triple quadrupole mass spectrometer was used in either positive ion mode or negative ion mode, depending on the characteristics of the analytes. The multiple reaction monitoring (MRM) mode was chosen for quantification (see the attached Table S1 for the selected masses for each of the pesticides) [39].

### 2.2. Target Screening of Risk Pesticide Residues and Parkinson’s Disease

#### 2.2.1. Target Prediction of Parkinson’s Disease

In order to identify potential disease targets closely related to PD, this study conducted searches using the keyword “Parkinson’s Disease” in three databases: GeneCards (https://www.genecards.org/, accessed on 5 June 2025), with a filtering criterion of Relevance Score > 20; the CTD database (https://ctdbase.org/, accessed on 5 June 2025), with a filtering criterion of Inference Score > 7; and MalaCards (www.malacards.org/, accessed on 5 June 2025), with a filtering criterion of Score > 20. Subsequently, the targets obtained from the databases were merged, and these targets were used for subsequent research analysis [40,41].

#### 2.2.2. Target Prediction of Toxic Substances

Target prediction of toxic substances: Using the SEA database (https://sea.bkslab.org/, accessed on 6 June 2025), CTD database (https://ctdbase.org/, accessed on 6 June 2025), and SwissTargetPrediction database (http://swisstargetprediction.ch/, accessed on 7 June 2025), we predicted the targets corresponding to toxic substances and calibrated the gene names using the Uniprot database (https://www.uniprot.org, accessed on 7 June 2025). Subsequently, we merged the targets obtained from the databases and eliminated duplicate entries. This process ultimately identified the component targets for further research [42,43].

#### 2.2.3. Intersection Gene Analysis

Intersection gene analysis: Import the potential toxic targets and PD predictive targets into Venny 2.1.0. A Venn diagram is created to identify potential toxic targets related to drugs and PD. Subsequently, the STRING database (https://string-db.org/, accessed on 9 June 2025) is used to analyze protein interactions to obtain a protein interaction topology file. Then, the protein–protein interaction (PPI) data is imported into Cytoscape 3.10.3 software to visually present the complex interaction network. Based on the intersection targets, the corresponding active ingredients are inferred, and a drug-component-target network diagram is generated using Cytoscape 3.10.3.

### 2.3. Pathway Enrichment Analysis of Target

This study utilized the Metascape database (https://metascape.org/gp/index.html, accessed on 10 June 2025) to perform Gene Ontology (GO) and Kyoto Encyclopedia of Genes and Genomes (KEGG) pathway enrichment analysis [44], in order to obtain the results of gene set enrichment. The statistical significance threshold for the enrichment analysis was set at p≤0.05. Finally, visualization was conducted using the microbioinformatics platform [45].

### 2.4. Molecular Docking

Molecular docking is an important tool in drug discovery as it enables us to understand molecular interactions; however, molecular interactions play a crucial role in all biological processes [46]. This study utilized the CB-Dock2 (https://cadd.labshare.cn/cb-dock2/php/index.php, accessed on 11 June 2025) platform to perform molecular docking analysis on the selected ligands and targets [47,48,49]. CB-Dock2 is a molecular docking tool based on AutoDock Vina analysis [50], which can automatically identify and analyze the binding sites of ligands and receptors. It simplifies the docking process while enhancing the accuracy of molecular docking. Compounds were imported into the CB-Dock2 platform to add hydrogen atoms and charges. Meanwhile, the crystal structure of the core target protein was obtained from the RCSB PDB (https://www.rcsb.org/, accessed on 15 June 2025) and processed in the CB-Dock2 platform to add hydrogen atoms and remove all water molecules. The binding affinity (kcal/mol) was calculated using CB-Dock2, and the energetically minimized optimal docking model was deduced. Molecular visualization is a key aspect for modeling, research analysis, and communication. Finally, the interaction details between the ligand and receptor were analyzed using PyMol v2.6 [51], and 3D interaction diagrams were generated, while 2D interaction diagrams were created using Discovery Studio (DS).

### 2.5. Molecular Dynamics Simulation

Molecular dynamics (MD) simulations of the protein-ligand complex obtained through molecular docking were performed for 100 ns using Gromacs v2022.03 [52]. The specific steps and parameter settings for the molecular dynamics simulation are as follows: (1) The pdb2gmx tool in Gromacs was used to convert the pdb file into a .gro format coordinate file, generating the corresponding topology file (.top) and force field parameter file, with the AMBER99SB-ILDN force field selected [53]. The small molecule was pre-processed using AmberTools22 software, adding the Generalized Amber Force Field (GAFF) to the small molecule [54]. Additionally, Gaussian 16W was utilized for hydrogen addition and RESP potential calculations for the small molecule, with the potential data incorporated into the topology file of the molecular dynamics system. (2) The three-point transferable intermolecular potential (TIP3P) solvent was chosen to solvate the protein-ligand complex, ensuring that the closest distance from the protein atoms to the edge of the water box was at least 1.2 nm [55], and an appropriate number of Na+ ions were added to neutralize the charge of the simulation system. (3) To achieve a stable system, the Steepest Descent algorithm is employed for energy minimization (EM) [56]; (4) For short-range electrostatic and van der Waals interactions, a cutoff distance of 1.0 nm is set, while the Particle Mesh Ewald (PME) method is used to compute long-range electrostatic interactions. The LINCS algorithm is utilized to constrain all involved hydrogen bonds; (5) During the MD simulation process, the Berendsen barostat is used to maintain a pressure of 1 bar, and the V-rescale temperature coupling method is employed to adjust the simulation temperature; (6) Subsequently, the protein structure undergoes a 100 ps pre-equilibration to optimize the initial conformation of the protein in the solvent. A 100 ps equilibrium is performed in the NVT ensemble to raise the temperature of the protein-solvent system to the target temperature. Then, a 100 ps equilibrium in the NPT ensemble is conducted for pressure equilibration of the solvent and protein system. (7) After completing energy minimization and system equilibration, a 100 ns molecular dynamics simulation was conducted for each protein-ligand complex. The simulation employed periodic boundary conditions and was performed under the isothermal-isobaric (NPT) ensemble, maintaining the system temperature at 300 K and pressure at 1 bar. The integration time step was set to 2 fs, with trajectory data (including coordinates, velocities, and energies) recorded every 10 ps for subsequent analysis.

## 3. Results and Discussion

### 3.1. Risk Pesticide Residues in Hawthorn

Researchers collected 18 fresh jujube samples from six counties and six locations in China (October 2023 and August 2024), and 56 hawthorn samples from three counties and sixteen locations (August to October 2023) (attached Table S2 for sample information). Subsequently, 101 pesticide residues in fresh jujube and hawthorn samples were detected using HPLC-MS/MS, allowing for the identification of pesticides posing certain risks. The detection limit of all tested pesticides can be considered as 0.05 mg/kg, and the pesticide residue detection method has good precision. At the same time, we submitted the recovery rates of each pesticide to demonstrate that the testing method has acceptable accuracy. Among the 101 pesticides tested, 38 kinds of pesticides were detected in the 18 fresh jujube samples. Each fresh jujube sample contained residues of different pesticides, with 8 pesticides exceeding the national maximum residue limits of China (Table 1). The chromatograms of mixed standard solutions with different concentrations (from 0.0050 mg/kg to 0.5000 mg/kg) and the screening results of 101 pesticides in these samples are shown in Figures S1–S8. The detection rates of phorate, deltamethrin, isofenphos-methyl, fenitrothion and abamectin in fresh jujube were the highest; 35 kinds of pesticides were detected in 56 hawthorn samples, and phorate was detected in each hawthorn. The residue of abamectin in one hawthorn sample (0.1143 mg/kg) was higher than the national limit standard (0.1000 mg/kg), and the over-standard rate of abamectin in 56 hawthorn samples was 1.79%. The pesticide residue data for fresh jujube and hawthorn samples are summarized in Table S3. Based on comprehensive consideration, phorate, deltamethrin, isofenphos-methyl, fenitrothion and abamectin were identified as risk residual pesticides in fresh jujube and hawthorn for subsequent network toxicology experiments.

**Table 1:** Typical 8 pesticides in fresh jujube samples.

Pesticide Name	Pesticide Type	MRL ^1^ (mg/kg)
phorate	insecticide	0.01
deltamethrin	insecticide	0.05
isofenphos-methyl	insecticide	0.01
fenitrothion	insecticide	0.50
Abamectin ^2^	insecticide	0.05
trichlorfon	insecticide	0.30
fenvalerate	insecticide	0.20
carbendazim	bactericide	0.50

^1^ MRL, maximum residue limit. ^2^ abamectin, in this manuscript, the term “abamectin” refers to abamectin B_1a_, which has the molecular formula R = -CH_2_CH_3_ B_1a_ C_48_H_72_O_14_ and a molecular weight of 873.09 MW.

### 3.2. Identification of Toxic Targets and Biological Mechanisms Related to Parkinson’s Disease

**Figure 2:**
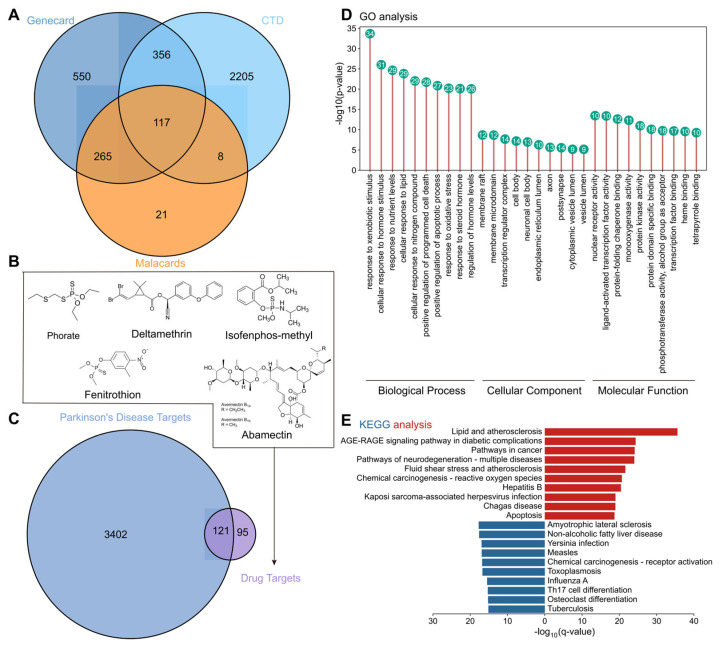
Identify the targets and biological mechanisms of toxicants associated with PD. (**A**) The Venn diagram of PD-related target intersection in GENECARD, CTD and malacads databases, (**B**) 2D structure of phosphate, deltamethrin, isofenphos-methyl, fenitrothion and abamectin toxicants, (**C**) Venn diagram of 121 PD target and toxicant target intersection, (**D**) GO analysis of 121 target intersection, (**E**) KEGG analysis of 121 target intersection.

The purpose of this study is to integrate the information from multiple databases to further explore the potential impact of the intersection targets and environmental toxicants related to PD. First, taking “Parkinson’s disease” as the keyword, 117 PD-related intersection targets were retrieved and screened in GENECARD, CTD and malacards databases, respectively (Figure 2A). These targets provide important clues for studying the pathological mechanism and potential therapeutic targets of PD. In order to further explore the impact of possible environmental toxicants from agricultural products on PD, the research team then predicted the potential targets of five pesticides and toxic substances, including phorate, deltamethrin, isofenphos-methyl, fenitrothion and abamectin, through the SEA database, CTD database and SwissTargetPrediction database. A total of 216 toxic targets were finally obtained (Figure 2B), which may play a role in the occurrence and development of PD. In order to identify the potential relationship between disease targets and toxic targets, the data of PD targets and toxic targets were imported into Venny 2.1.0 software for intersection analysis. Venn diagram analysis showed that there was significant overlap between these two types of targets, and 121 poison-related PD intersection targets were finally obtained (Figure 2C). The action targets of the five pesticides have a certain degree of overlap with PD disease-related targets, which provides a deeper understanding of the relationship between environmental toxins and PD, and provides possible targets for future drug development.

### 3.3. GO Enrichment and KEGG Pathway Analysis

Subsequently, this study used the metascape platform to conduct gene ontology (GO) analysis and KEGG pathway enrichment analysis on the 121 intersection targets, in order to reveal the potential role of these targets in biological processes, cellular components and molecular functions. GO analysis results (Figure 2D) show that these intersecting targets are involved in many important biological processes, especially response to xenobiotic stimulus, cellular response to hormone stimulus, response to nutrients levels, cellular response to lipid, which are closely related to the pathological process of PD, and may involve the damage of neurons, the destruction of cell membrane structure and other mechanisms. In addition, KEGG pathway analysis (Figure 2E) revealed several important metabolic pathways, such as lipid metabolism and oxidative stress response, which are closely related to the pathogenesis of PD. Especially, the AGE-RAGE signaling pathway, lipid metabolism pathway and oxidative stress pathway play an important role in the process of neurodegenerative diseases.

GO enrichment and KEGG pathway analysis have provided the macro-level perspective on the potential pathophysiological effects of pesticide exposure. The significantly enriched terms collectively implicate core mechanisms involving oxidative stress and programmed cell death. These results strongly suggest that residual pesticides present in red dates and hawthorn can disrupt intracellular redox balance, ultimately leading to oxidative damage and apoptosis of neurons—particularly vulnerable dopaminergic neurons—reflecting a well-established pathway in the pathogenesis of PD. Furthermore, enrichment of terms associated with lipid metabolism pathways (cellular response to lipid, lipid and atherosclerosis) indicates that pesticide-induced perturbations extend beyond oxidative stress to include dysregulation of lipid homeostasis. Given the critical importance of neuronal membrane lipid composition in maintaining cellular function, disturbances in lipid metabolism are closely linked to the pathogenesis of neurological disorders, including PD. To sum up, this study revealed the possible relationship between PD and environmental toxicants by integrating target data from various databases, and further clarified its potential molecular mechanism through GO and KEGG analysis. These findings provide new ideas for the pathological study of PD and the discovery of drug targets, and provide a theoretical basis for the development of new treatment strategies.

### 3.4. Construction of Enrichment Analysis Network Diagram and Drug-Disease Target Network Diagram

**Figure 3:**
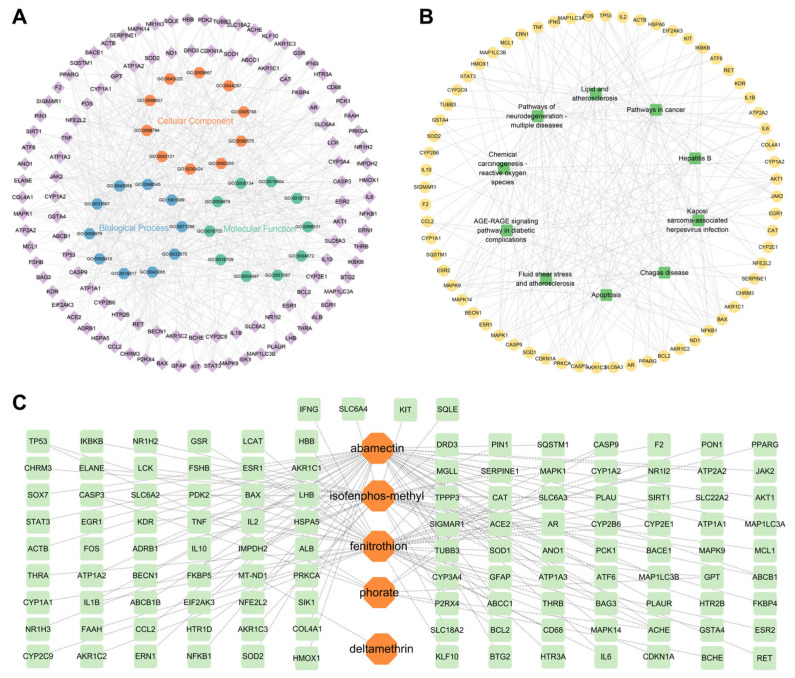
Network diagram construction. (**A**) The interactions between PD-related targets are classified according to Gene Ontology (GO) terminology. Targets are divided into three categories: biological process (blue), cell composition (orange) and molecular function (green). (**B**) Network analysis shows the enrichment KEGG pathway of 121 PD-related targets, including the AGE-RAGE signaling pathway, lipid metabolism and atherosclerosis, neurodegenerative diseases, cancer and other related pathways. (**C**) The molecular interaction network shows the relationship between environmental toxins and their corresponding targets. The hexagonal shape of the color block represents environmental toxins, and the surrounding network displays corresponding biological targets and related molecular pathways.

This study selected and extracted the top 30 genes from GO (BP:CC:MF = 10:10:10), the top 10 KEGG and their corresponding entries, and KEGG relationship pairs. A network diagram of biological processes/KEGG signaling pathway key target interactions was constructed using Cytoscape software. The GO network diagram consists of 140 nodes and 530 edges (Figure 3A), while the KEGG network diagram consists of 74 nodes and 209 edges (Figure 3B). Subsequently, based on 121 intersecting targets, this study used Cytoscape software to construct a toxin (phorate, deltamethrin, isofenphos-methyl, fenitrothion, and abamectin) intersecting target network diagram (Figure 3C). These toxins have been shown to have neurotoxicity and may promote the occurrence or progression of PD through interactions with specific proteins and signaling pathways. By linking environmental toxins with disease-related molecular targets, this analysis emphasizes the importance of environmental factors in the pathogenesis of PD, providing a new perspective for future biomarker discovery and treatment target research.

Drug disease-target network diagram constructed by the research institute not only intuitively demonstrates the potential multi-component and multi-target characteristics of fresh jujube and hawthorn in intervening PD, but also provides important clues for understanding their possible mechanisms of action from a systems biology perspective. Firstly, network topology analysis reveals key targets and their core positions. The core targets identified in this study are widely involved in key biological processes, which are highly consistent with the known pathological mechanisms of PD. This suggests that residual pesticides in jujube and hawthorn may affect the occurrence of PD by synergistically acting on these core nodes. Meanwhile, GO enrichment and KEGG pathway enrichment analysis map discrete targets to organic functional modules. The analysis results go beyond the limitations of a single target and reveal that pesticide residue-induced PD is a systemic problem involving multiple pathways and functions. Overall, these panels reveal the complex interplay of genetic susceptibility, environmental exposure, and disease mechanisms in PD by integrating multi-level data, including genetics, molecular mechanisms, and environmental factors.

### 3.5. Screening Pesticide Targets Related to Parkinson’s Disease

**Figure 4:**
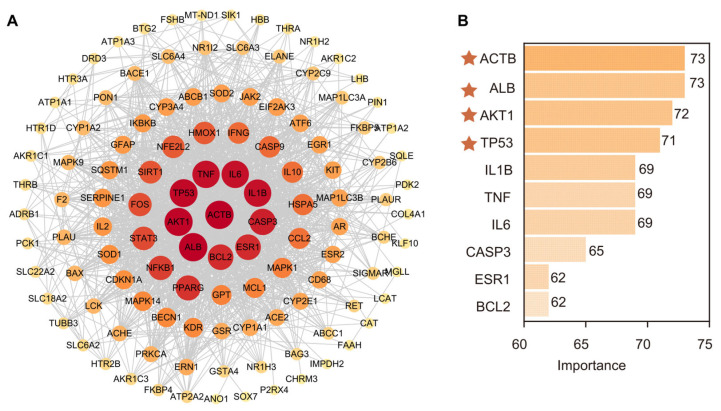
Key target screening. (**A**) 121 intersecting target PPI network diagrams, (**B**) ranking of the top 10 target degrees.

The PPI network diagram visualizes the protein–protein interaction network of 121 PD-related targets, with a focus on showcasing the interrelationships among these targets. The central nodes (represented in deep red), such as TP53, TNF, IL6, AKT1, and ACTB (Figure 4A), are particularly associated with the pathophysiology of PD and involve key biological processes such as cell apoptosis, inflammation, and oxidative stress. These targets are interconnected through complex signaling networks and may play a role in neurodegenerative diseases and cell damage in PD. The bar chart displays the “degree” quantitative ranking of the top 10 targets (Figure 4B), reflecting their centrality and degree of interaction in the network. ACTB, ALB, AKT1, and TP53 rank high, indicating that they play a critical role in the PD molecular pathway. These targets are involved in basic processes such as cell cycle regulation, survival, and apoptosis, which are disrupted in PD. Identifying these key targets is of great significance for understanding the molecular mechanisms of PD and identifying potential therapeutic targets to mitigate the impact of environmental toxins on the disease.

### 3.6. Molecular Docking Analysis

Molecular docking is an important tool for drug discovery as it enables us to understand molecular interactions, which play a crucial role in all biological processes. Download the ‘.sdf’ files of phorate, deltamethrin, isofenphos-methyl, fenitrotion, and abamectin from the Pubchem database, and download the crystal structures of ACTB, AKT1, ALB, and TP53 from the RSCB PDB database. Subsequently, this study utilized the Cb-Dock2 platform for molecular docking analysis.

The Vina score reflects the binding energy between proteins and small molecules, and is typically used to evaluate the stability of molecular docking. Negative values indicate strong binding affinity, and the lower the binding energy, the more stable the binding. A positive value indicates weak binding and may even result in unstable binding. According to the Vina score (Table 2), abamectin exhibits the strongest binding affinity among all target proteins, especially with the strongest binding ability to ALB. phorate shows relatively weak binding affinity, especially with almost ineffective binding to ACTB and TP53. Overall, most toxins have negative binding to ACTB, AKT1, ALB, and TP53, indicating that these toxins may affect the occurrence and development of PD by interfering with the function of these targets. These results provide potential targets for future research, which may help develop new treatment strategies or preventive measures.

**Figure 5:**
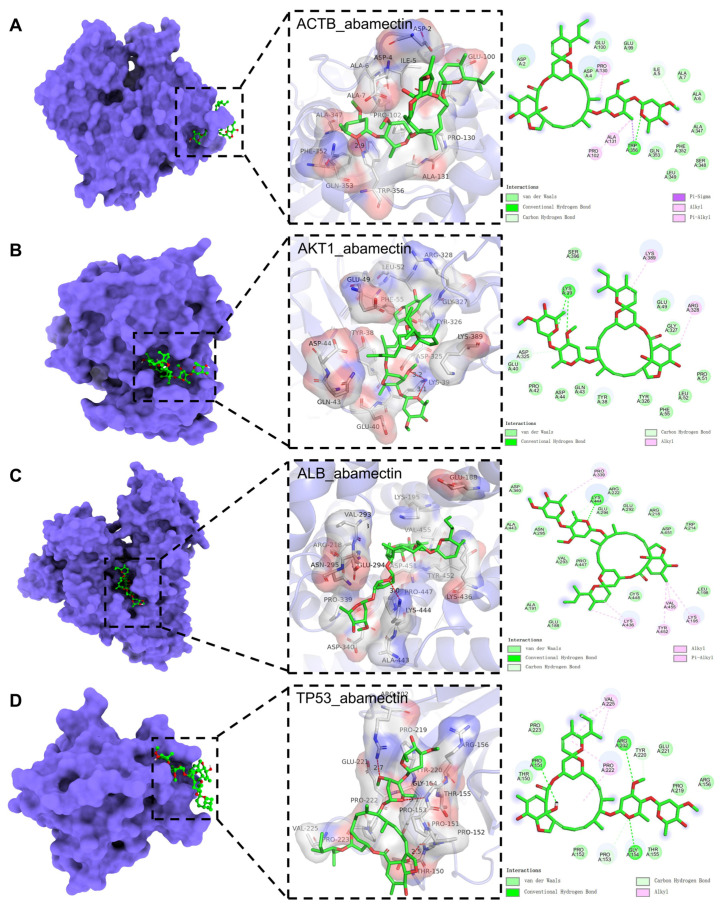
Molecular docking results of abamectin and the targets. (**A**) Docking model of ACTB-abamectin with the lowest binding affinity (−8.1 kcal/mol). (**B**) Docking model of AKT1-abamectin with the lowest binding affinity (−8.2 kcal/mol). (**C**) Docking model of ALB-abamectin with the lowest binding affinity (−10.2 kcal/mol). (**D**) Docking model of TP53-abamectin with the lowest binding affinity (−8.6 kcal/mol).

Subsequently, DS and PyMol were used to analyze the interactions between abamectin and ACTB, AKT1, ALB, and TP53, as shown in Figure 5A. Abamectin formed hydrogen bonds and Pi-Sigma bonds (π-σ) with TRP356 of ACTB, hydrophobic interactions with PRO130, PRO102, and ALA131, carbon–hydrogen bonds with ILE5, and van der Waals forces with surrounding amino acids. As shown in Figure 5B, abamectin forms hydrogen bonds with LYS39 of AKT1, hydrophobic interactions with ARG328 and LYS389, and van der Waals forces with other surrounding amino acids. As shown in Figure 5C, abamectin forms hydrogen bonds with LYS444 of ALB, hydrophobic interactions with PRO339, LYS436, TYR452, VAL455, and LYS195, and van der Waals forces with other surrounding amino acids. As shown in Figure 5D, abamectin forms hydrogen bonds with ARG202, GLY154, and PRO151 of TP53, carbon–hydrogen bonds with PRO153 and TYR220, hydrophobic interactions with PRO22 and VAL225, and van der Waals forces with other surrounding amino acids.

### 3.7. Molecular Dynamics Simulation Reveals the Stability of the Complex

Based on the results of molecular docking, this study conducted 100 ns molecular dynamics simulation analysis on the complexes of ACTB abamectin, AKT1 abamectin, ALB abamectin, and TP53 abamectin. Molecular dynamics simulation indicators include RMSD, RMSF, Rg, and hydrogen bonding. RMSD is used to evaluate the accuracy of protein structure prediction and is an important basis for measuring the stability of the system [57]. The lower the RMSD value, the higher the protein ligand stability [58]. RMSF is another important parameter in molecular dynamics simulations, whose value represents the fluctuation amplitude of amino acid residues deviating from their initial positions throughout the simulation system [59]. The RMSF value describes the degree of change in the configuration of a molecule over time relative to its initial structure, and is an important indicator of particle mobility. In the trajectory, the value of RMSF is determined by the flexibility of the peptide chain and the impact of the environment on the protein molecule. Rg is the root mean square distance of the center of mass defined relative to the rotational coordinate axis for all atoms of a large molecule [60]. Rg is a physical quantity used to describe the tightness of protein structure. Proteins with relatively stable structures have better tightness, and the corresponding Rg value will be smaller [61]. Hydrogen bonding, as a strong noncovalent interaction, plays a role in promoting the interaction between protein surface atoms and solvent molecules [62], and helps ligand binding to identify precise interactions with active sites [63]. Therefore, the number of hydrogen bonds is also used to determine the stability of protein ligand complexes in simulation processes.

#### 3.7.1. Stability Analysis of ACTB_Abamectin Complex

**Figure 6:**
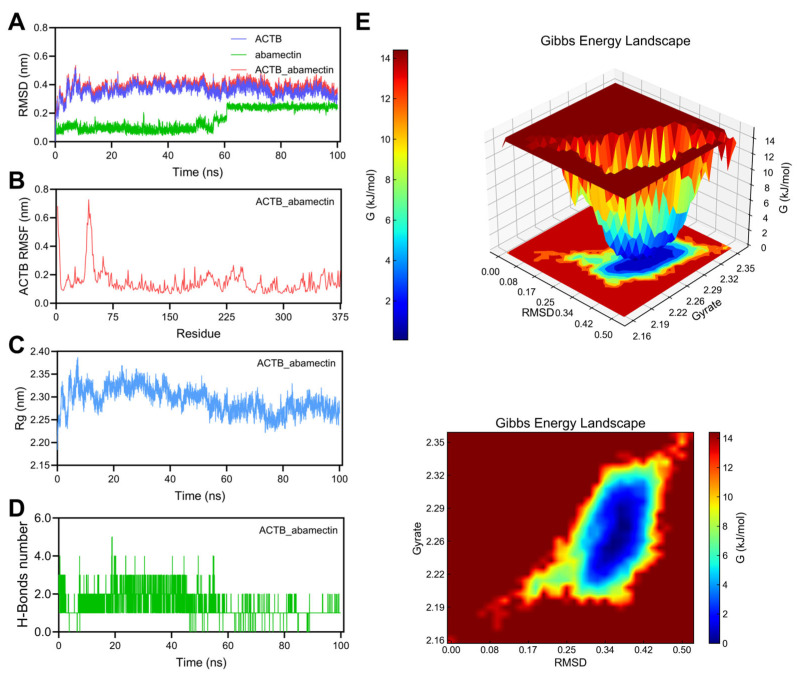
100 ns MD simulation of ACTB_abamectin complex. (**A**) RMSD curve of ACTB (purple line), abamectin (green line), and the ACTB_abamectin complex (red line). (**B**) RMSF curve of ACTB. (**C**) Rg curve of ACTB. (**D**) Hydrogen bonds of the ACTB_abamectin complex. (**E**) Three-dimensional and two-dimensional Gibbs free energy landscape of the ACTB_abamectin complex.

As shown in Figure 6A, the trend of RMSD of ACTB is basically consistent with that of the complex. Abamectin’s RMSD shows relatively large structural fluctuations, but maintains a certain degree of stability. The RMSD of the complex formed by the binding of ACTB and abamectin showed small fluctuations, with a range of RMSD values from approximately 0.2 nm to 0.5 nm, indicating that the structure of the complex is relatively stable. As shown in Figure 6B, after binding with abamectin, the RMSF values of some residues of the ACTB protein are relatively high, reaching about 0.7 nm, indicating that these regions have significant dynamic fluctuations in the complex and may have high flexibility during the binding process. As shown in Figure 6C, the Rg value is approximately between 2.25 nm and 2.35 nm. This indicates that the molecular structure of the complex remains stable without significant expansion or contraction, suggesting that the size and shape of the complex remain consistent in solution. As shown in Figure 6D, the number of hydrogen bonds fluctuates during the simulation process, with a maximum of 5 hydrogen bonds and a minimum of 1 hydrogen bond. This wave of activity shows that the formation and breaking of hydrogen bonds during the binding process of the complex with abamectin have an impact on its stability. The Gibbs free energy morphology diagram (Figure 6E) shows that in the range of RMSD of approximately 0.25–0.42 nm and Rg value of approximately 2.19–2.32 nm, the composite exhibits lower free energy values, indicating that the composite is most stable in this region and has a lower free energy state.

ACTB is a protein widely present in human cells and plays a crucial role in the formation of the cytoskeleton. It is closely related to the maintenance, movement, and integration with the environment of cells [64]. In clinical medicine, abnormalities in the ACTB protein are associated with various diseases, such as diffuse large B-cell lymphoma, developmental abnormalities, adolescent-onset dystonia, and PD [65]. In recent years, the ACTB protein has also become an important target for drug development. Researchers are exploring the use of nanotechnology and other methods to deliver ACTB protein-related drugs to the brain for the treatment of neurological diseases such as PD. It has been found that actin can induce mitochondrial transplantation (MT), increase the rate of mitochondrial biosynthesis in PD mice, and increase the number of mitochondrial subunits, thereby alleviating PD [66]. According to MD simulations, abamectin may enhance the progression of PD.

#### 3.7.2. Stability Analysis of AKT1_Abamectin Complex

**Figure 7:**
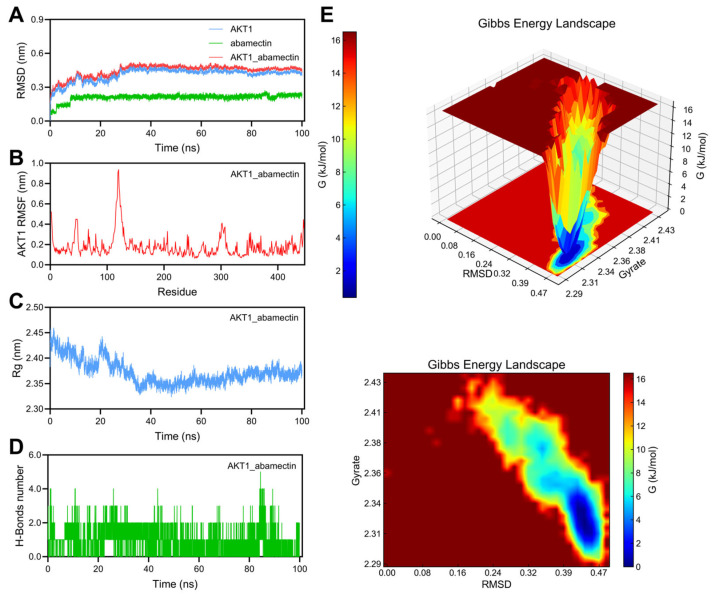
100 ns MD simulation of AKT1_abamectin complex. (**A**) RMSD curve of AKT1 (purple line), abamectin (green line), and the AKT1_abamectin complex (red line). (**B**) RMSF curve of AKT1. (**C**) Rg curve of AKT1. (**D**) Hydrogen bonds of the AKT1_abamectin complex. (**E**) Three-dimensional and two-dimensional Gibbs free energy landscape of the AKT1_abamectin complex.

As shown in Figure 7A, the RMSD fluctuation trend of the AKT1 protein is still consistent with the trend of the complex. Abamectin exhibits certain RMSD fluctuations, ranging from 0.2 nm to 0.25 nm. The RMSD fluctuation range of AKT1_abamectin complex is between 0.4 nm and 0.5 nm, indicating that the complex has good structural stability. As shown in Figure 7B, the residues of AKT1 fluctuate after binding with Abamectin, and some residues have larger fluctuations in the complex, especially those in the range of 100 to 150, with RMSF values close to 1 nm. This indicates that these regions may have higher flexibility and may participate in drug binding or interactions. As shown in Figure 7C, the Rg value is roughly between 2.35 and 2.45 nm, indicating that the size of the complex is relatively stable during the simulation process and no significant shrinkage or expansion has occurred. As shown in Figure 7D, during the simulation process, the number of hydrogen bonds exhibited significant fluctuations, with a maximum of up to 5 hydrogen bonds and a minimum of nearly 0 hydrogen bonds. This fluctuation may reflect the dynamic changes in the binding strength of the complex during the binding process with abamectin. The Gibbs free energy morphology diagram (Figure 7E) indicates that the lower free energy region is located between RMSD values of approximately 0.39–0.45 nm and Rg values of approximately 2.30–2.34 nm. This region exhibits lower free energy, indicating that the complex is stable in this area and has lower binding energy.

AKT1 is a key molecule in the cellular signaling pathway, involved in regulating various processes such as cell growth, survival, metabolism, and differentiation. The study of recombinant AKT1 protein provides valuable insights into its role in various diseases, particularly in the research of cancer, metabolic diseases, and degenerative diseases [67]. Natural medicines can activate the PI3K/AKT signaling pathway, regulate the expression of apoptosis factors such as Bcl-2 and Bax, significantly reduce 6-OHDA-induced apoptosis of SH-SY5Y cells, and thus treat PD [68]. Network pharmacology combined with experimental verification shows that apigenin, as the active ingredient of scallion flowers, inhibits the PI3K/AKT/NF-κB pathway to combat PD [69]. Through MD simulation, it was found that abamectin has a good binding ability with AKT1, indicating that abamectin has a risk of causing PD.

#### 3.7.3. Stability Analysis of ALB_Abamectin Complex

**Figure 8:**
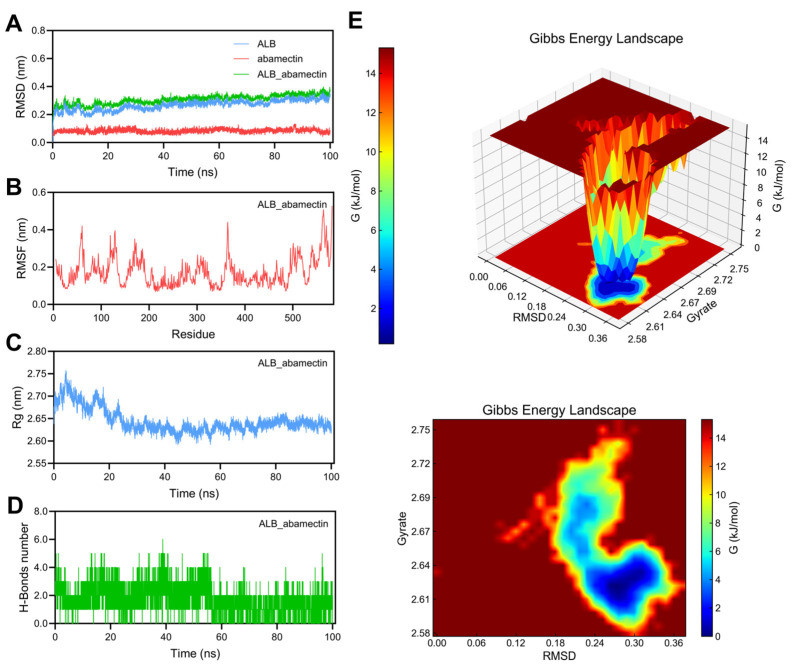
100 ns MD simulation of ALB_abamectin complex. (**A**) RMSD curve of ALB (blue line), abamectin (red line), and the ALB_abamectin complex (green line). (**B**) RMSF curve of ALB. (**C**) Rg curve of ALB. (**D**) Hydrogen bonds of the ALB_abamectin complex. (**E**) Three-dimensional and two-dimensional Gibbs free energy landscape of the ALB_abamectin complex.

As shown in Figure 8A, the RMSD of the ALB protein is slightly lower than that of the complex, and the RMSD value range of abamectin is about 0.08–0.1 nm, showing good stability. However, compared to the ALB protein, abamectin has smaller fluctuations. The RMSD value of the ALB_abamectin complex is between 0.2 nm and 0.4 nm, which is relatively stable, indicating that Abamectin can also maintain considerable stability after binding with ALB. As shown in Figure 8B, certain residues in the complex (especially around positions 100 to 350) exhibit significant fluctuations, with RMSF values exceeding 0.4 nm. These regions may be involved in drug molecule binding or have higher flexibility. This fluctuation indicates that the flexibility of certain regions may be a key factor in drug protein interactions during the binding process. As shown in Figure 8C, the Rg value is approximately between 2.60 and 2.75 nm, indicating that the complex maintained a relatively stable structural size during the simulation period. A smaller fluctuation means that the composite as a whole has not undergone significant expansion or contraction, maintaining a relatively stable spatial structure. As shown in Figure 8D, the fluctuation range of the number of hydrogen bonds is approximately between 2 and 6 hydrogen bonds. The stability and formation of hydrogen bonds are crucial for the bonding strength of the complex. The figure shows that there is a certain fluctuation in the number of hydrogen bonds in the complex during the simulation process, indicating that the stability of the complex is dynamically affected by hydrogen bonds. The Gibbs free energy morphology diagram (Figure 8E) shows that the lower free energy region is located between RMSD values of 0.24–0.34 nm and Rg values of 2.60–2.64 nm, indicating that the binding of the complex is thermodynamically stable and has the lowest energy within this range.

ALB is a non-glycoprotein with high structural stability. It can bind with various endogenous and exogenous substances, including lipids, hormones, vitamins, and drugs, thereby playing a role in transportation and storage in the body. In addition, it also participates in the transportation of various drugs and hormones, regulating their distribution and metabolism in the body. Some studies suggest that ALB in plasma indicates a decline in cognitive ability in PD [70,71]. The ratio of red blood cell distribution width to albumin (RAR) is an indicator of systemic inflammation and nutritional status. Clinical studies have shown a positive non-linear correlation between RAR and the incidence of PD, and when RAR exceeds approximately 3.12, the risk of PD significantly increases and continues to rise [72]. The strong binding and good kinetic index of abamectin to ALB highlight the potential of this pesticide toxic pollutant to induce PD.

#### 3.7.4. Stability Analysis of TP53_Abametin Complex

**Figure 9:**
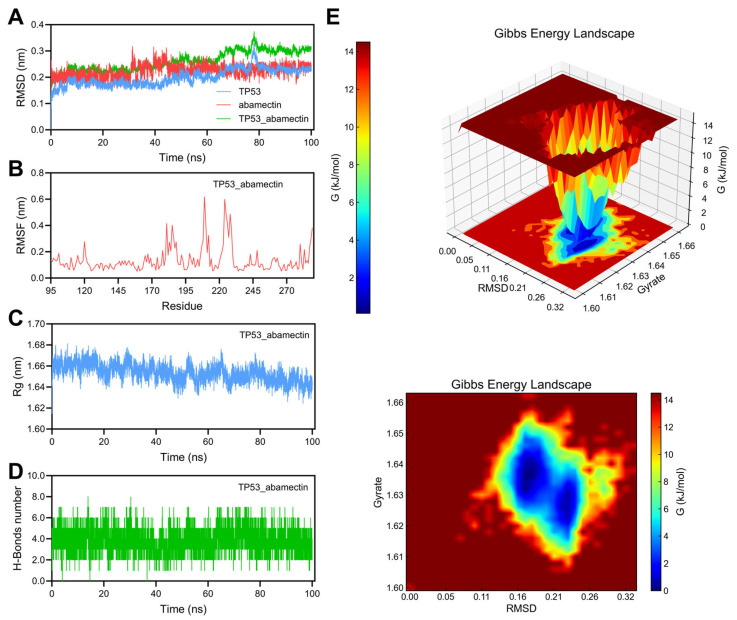
100 ns MD simulation of TP53_abamectin complex. (**A**) RMSD curve of TP53 (blue line), abamectin (red line), and the TP53_abamectin complex (green line). (**B**) RMSF curve of TP53. (**C**) Rg curve of TP53. (**D**) Hydrogen bonds of the TP53_abamectin complex. (**E**) Three-dimensional and two-dimensional Gibbs free energy landscape of the TP53_abamectin complex.

As shown in Figure 9A, the RMSD fluctuation of the TP53 protein during the simulation process was relatively small, ranging from 0.15 nm to 0.3 nm, indicating that the structure of TP53 maintained high stability during the simulation process. The RMSD of abamectin fluctuates significantly, ranging from 0.15 nm to 0.33 nm, indicating a certain degree of instability. The RMSD of TP53 Abamectin is higher, showing lower structural stability than TP53 or Abamectin alone. As shown in Figure 9B, after binding to Abamectin, the TP53 protein exhibits significant fluctuations mainly concentrated at positions 170 to 230, with RMSF values ranging from 0.4 nm to 0.6 nm. This indicates that these residues have high flexibility and may be associated with Abamectin binding or play an important role in protein conformational changes. As shown in Figure 9C, the Rg value is approximately between 1.63 nm and 1.68 nm. This range indicates that the composite did not undergo significant expansion or contraction during the simulation process and maintained a stable molecular shape and structure as a whole. As shown in Figure 9D, the number of hydrogen bonds fluctuates between 4 and 8. The fluctuation in the number of hydrogen bonds reflects the change in the stability of the complex structure. Although the fluctuation is significant, the formation of hydrogen bonds plays an important role in maintaining the stability of the complex at certain moments. The Gibbs free energy morphology diagram (Figure 9E) shows that the lower free energy region is located between RMSD values of 0.15–0.24 nm, and Rg values are approximately between 1.62 and 1.65 nm, indicating that the composite is thermodynamically stable within this range.

TP53 is involved in regulating various cellular processes, including cell cycle arrest, autophagy, aging, apoptosis, DNA repair, mitochondrial metabolism, and redox homeostasis [73]. Research has shown that in cellular and animal models of PD, as well as in the brains of PD patients, TP53 levels and activity are significantly increased in affected neurons [74]. The response of TP53 to neurodegenerative stress is closely related to the degeneration of dopaminergic neurons, accompanied by mitochondrial dysfunction, production of reactive oxygen species (ROS), abnormal protein aggregation, and autophagy damage, thereby inducing the degeneration of dopaminergic neurons and leading to the occurrence of PD [75].

## 4. Conclusions

This study used UPLC-MS/MS technology to screen for pesticide residues in hawthorn and fresh jujube, identifying five high-risk pesticides (phorate, deltamethrin, isofenphos-methyl, fenitrothion, and abamectin), and comprehensively applying network toxicology, molecular docking, and molecular dynamics simulation methods to systematically explore their potential effects on Parkinson’s disease (PD). In total, 117 PD-related targets and 216 potential pesticide action targets were obtained through joint screening of multiple databases, and 121 common action targets were identified through cross-analysis. GO and KEGG enrichment analysis showed that these targets are significantly involved in biological processes such as cell apoptosis, inflammatory response, and oxidative stress, and are closely related to the pathological mechanism of PD. Further construction of toxin target pathway interaction networks and PPI networks revealed that core targets such as TP53, AKT1, and ACTB play a critical role in the occurrence and development of PD. The molecular docking results showed a strong binding affinity between the selected pesticide and the aforementioned core targets, and molecular dynamics simulations further validated the stability of its complex system.

Research has shown that five pesticides may intervene in PD-related molecular mechanisms by regulating key targets and signaling pathways, thereby posing potential neurotoxic risks. This study provides a theoretical basis for elucidating the association between pesticide exposure and the occurrence of Parkinson’s disease, and lays the foundation for subsequent in vivo and in vitro experiments and risk assessment research. The results suggest that the potential impact of pesticide residues in the daily diet on neurodegenerative diseases should be taken seriously, and it is recommended to further strengthen the supervision and health risk prevention of pesticide use in agricultural products. In the future, we will continue to investigate the neurotoxic effects of pesticide exposure using biological samples, with a particular focus on neurodegenerative disorders such as Parkinson’s disease and Alzheimer’s disease. More comprehensive and scientifically substantiated findings will be elucidated through studies employing cellular and animal models in the near future.

## Figures and Tables

**Table 2:** Molecular Docking Results.

Protein	PDB ID	Vina Score (kcal/mol)				
		Abamectin	Deltamethrin	Fenitrothion	Isofenphos-Methyl	Phorate
ACTB	6NBW	−8.1	−7.0	−6.1	−6.4	−3.9
AKT1	3O96	−8.2	−9.9	−6.8	−7.1	−4.3
ALB	1BM0	−10.2	−9.1	−6.0	−6.3	−4.8
TP53	1TSR	−8.6	−8.6	−6.1	−5.9	4.2

## Data Availability

The original contributions presented in this study are included in the article/Supplementary Materials. Further inquiries can be directed to the corresponding authors.

## Supplementary Materials

The following supporting information can be downloaded at: https://www.mdpi.com/article/10.3390/foods14193324/s1, Figure S1. Positive mode chromatogram during pesticide residue detection the mixed standard prepared with blank fresh jujube sample as matrix; Figure S2. Negative mode chromatogram during pesticide residue detection the mixed standard prepared with blank fresh jujube sample as matrix; Figure S3. Positive mode chromatogram during pesticide residue detection the mixed standard prepared with blank hawthorn sample as matrix; Figure S4. Negative mode chromatogram during pesticide residue detection the mixed standard prepared with blank hawthorn sample as matrix; Figure S5. Positive mode chromatogram in pesticide residue detection of fresh jujube samples; Figure S6. Negative mode chromatogram in pesticide residue detection of fresh jujube samples; Figure S7. Positive mode chromatogram in pesticide residue detection of hawthorn samples; Figure S8. Negative mode chromatogram in pesticide residue detection of hawthorn samples; Table S1: The selected masses for each of the pesticides; Table S2: Sample information; Table S3: Pesticide residues in fresh jujube and hawthorn samples.

## Abbreviations

The following abbreviations are used in this manuscript:

PD	Parkinson’s Disease
UPLC-MS/MS	Ultra Performance Liquid Chromatography–Tandem Mass Spectrometry
PPI	Protein–Protein Interaction
GO	Gene Ontology
KEGG	Kyoto Encyclopedia of Genes and Genomes
DS	Discovery Studio
GAFF	Generalized Amber Force Field
TIP3P	Transferable Intermolecular Potential
EM	Energy Minimization
PME	Particle Mesh Ewald
MD	Molecular Dynamics
