# Supplementary material for: Speculation on the Mechanism of Parkinson’s Disease Induced by Risk Residual Pesticides in Fresh Jujube and Hawthorn Through Network Toxicology and Molecular Docking Analysis
Source: Foods. 2025 Sep 25;14(19):3324. doi: 10.3390/foods14193324 (PMC12524234; doi:10.3390/foods14193324)
Supplement: Supplementary file 1 [file foods-14-03324-s001.zip › Supplementary materials.pdf]

Speculation on the mechanism of Parkinson's disease induced by risk residual pesticides in Fresh jujube and Hawthorn through network toxicology and molecular docking analysis

Supplementary materials

Contents：

Figure.S1 Positive mode chromatogram during pesticide residue detection the mixed standard prepared with blank fresh jujube sample as matrix.············2

Figure.S2 Negative mode chromatogram during pesticide residue detection the mixed standard prepared with blank fresh jujube sample as matrix.············3

Figure.S3 Positive mode chromatogram during pesticide residue detection the mixed standard prepared with blank hawthorn sample as matrix.············4

Figure.S4 Negative mode chromatogram during pesticide residue detection the mixed standard prepared with blank hawthorn sample as matrix.············5

Figure.S5 Positive mode chromatogram in pesticide residue detection of fresh jujube samples.············6

Figure.S6 Negative mode chromatogram in pesticide residue detection of fresh jujube samples.············7

Figure.S7 Positive mode chromatogram in pesticide residue detection of hawthorn samples.············8

Figure.S8 Negative mode chromatogram in pesticide residue detection of hawthorn samples.············9

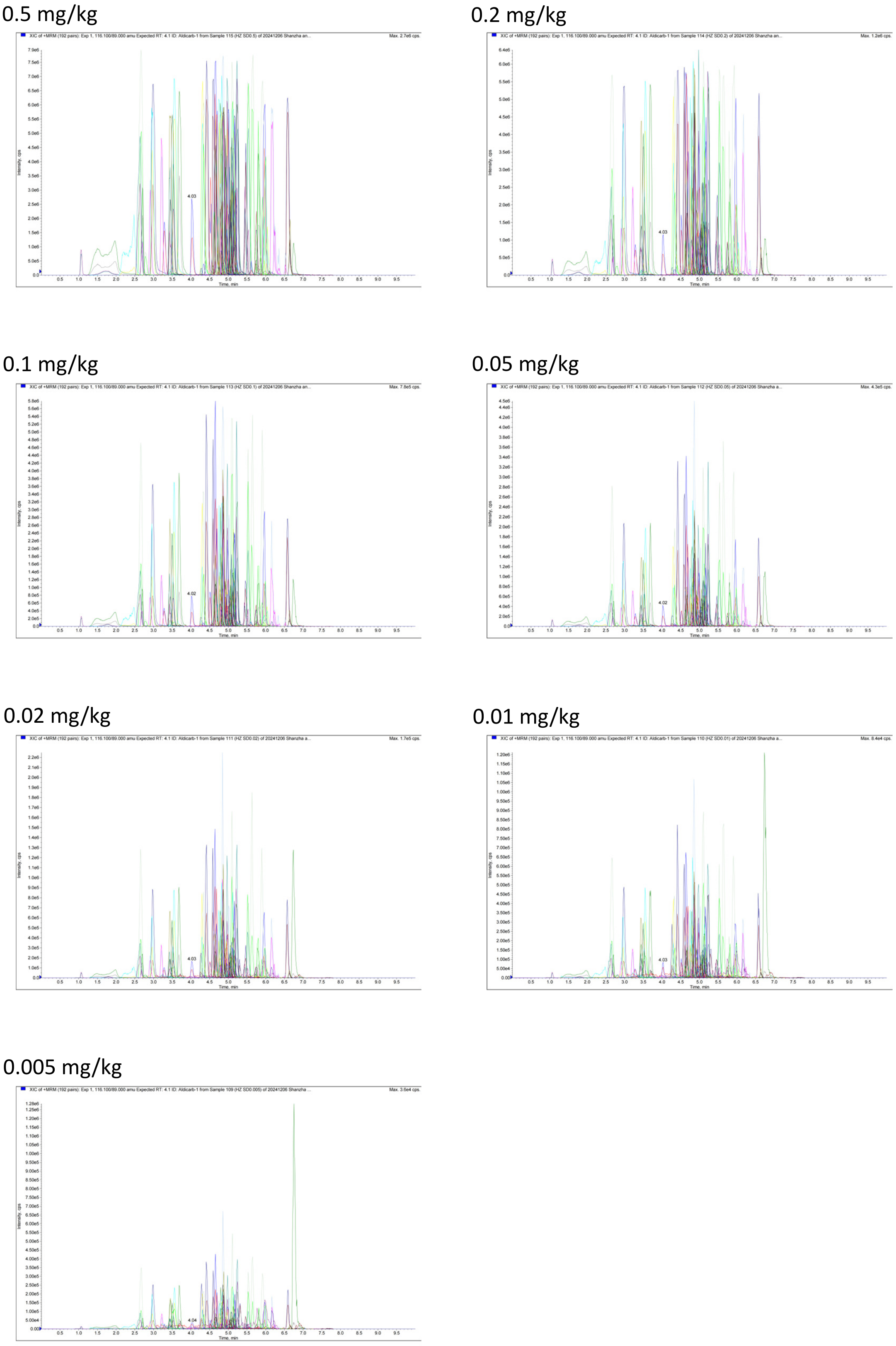

Figure.S1 Positive mode chromatogram during pesticide residue detection the mixed standard prepared with blank fresh jujube sample as matrix.

0.5 mg/kg

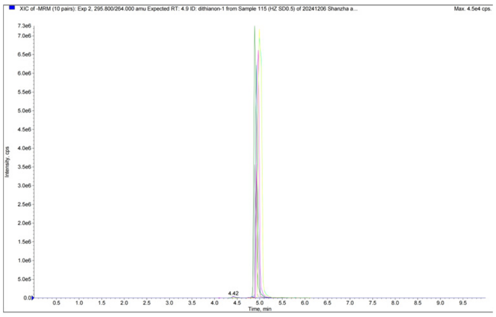

0.2 mg/kg

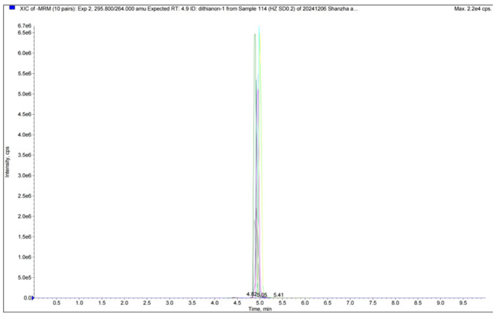

0.1 mg/kg

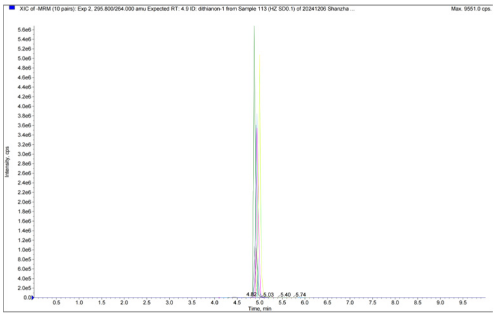

0.05 mg/kg

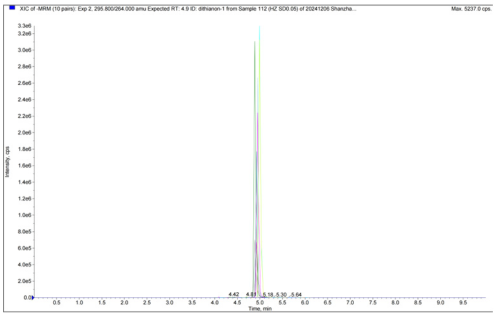

0.02 mg/kg

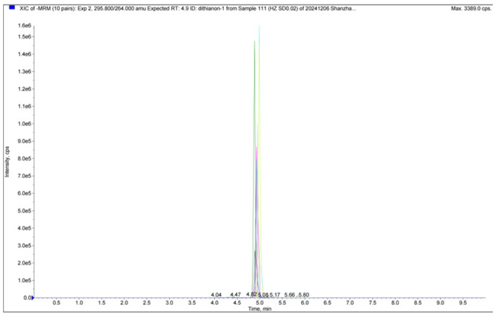

0.01 mg/kg

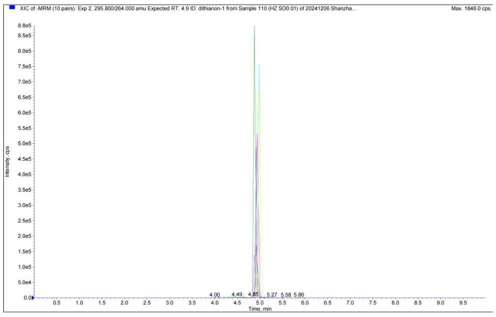

0.005 mg/kg

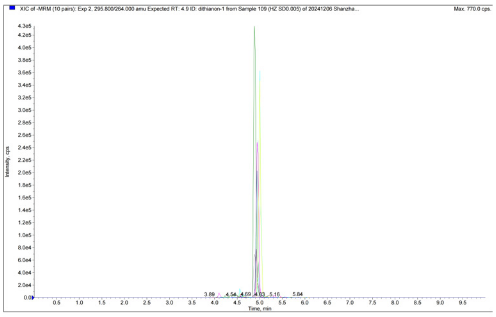

Figure.S2 Negative mode chromatogram during pesticide residue detection the mixed standard prepared with blank fresh jujube sample as matrix.

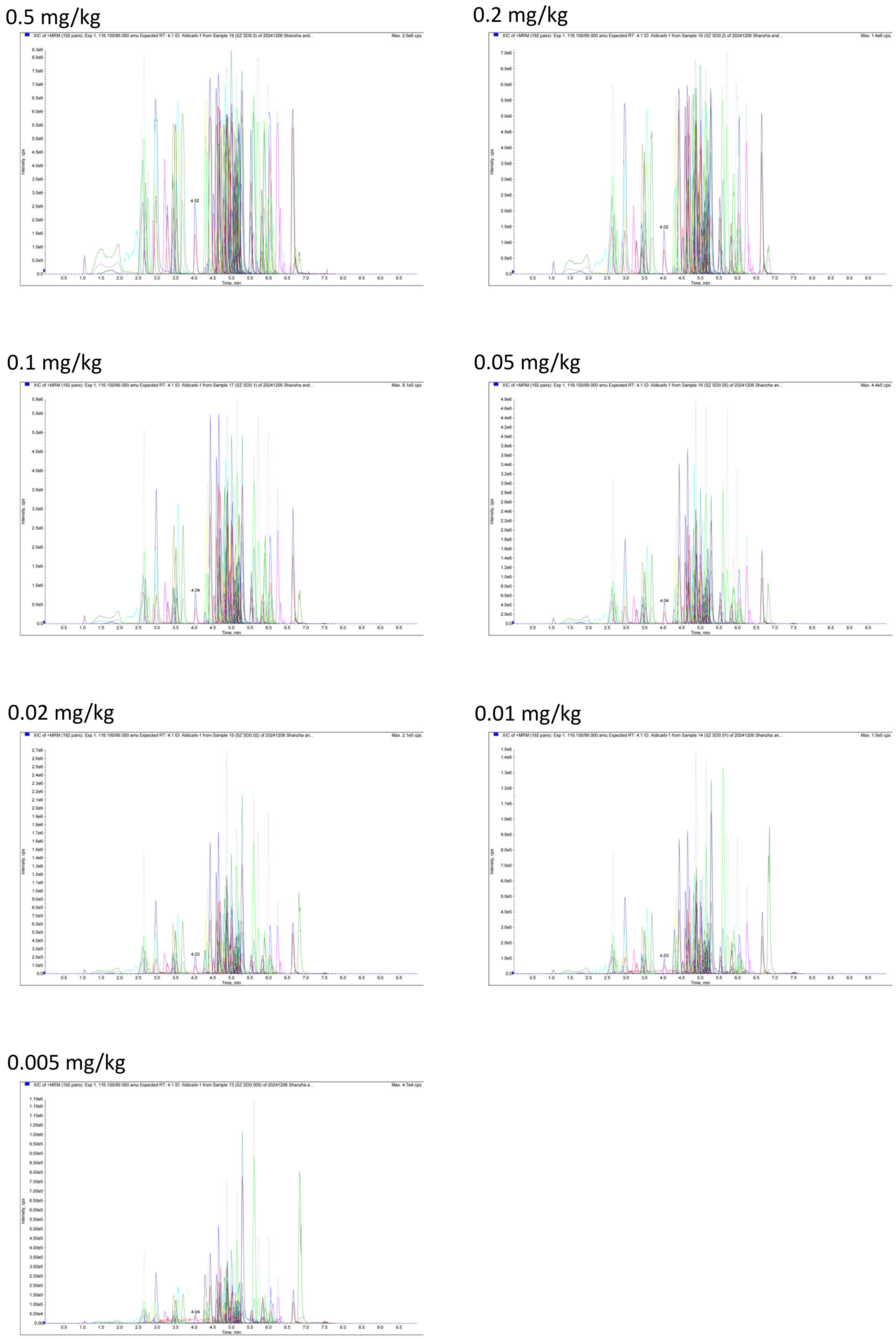

Figure.S3 Positive mode chromatogram during pesticide residue detection the mixed standard prepared with blank hawthorn sample as matrix.

0.5 mg/kg

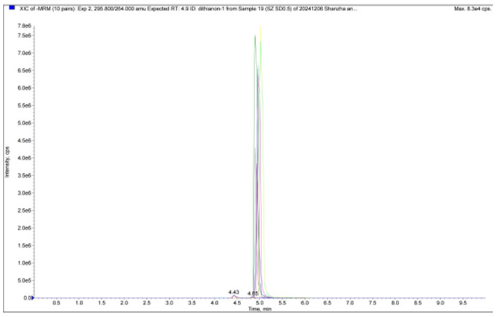

0.2 mg/kg

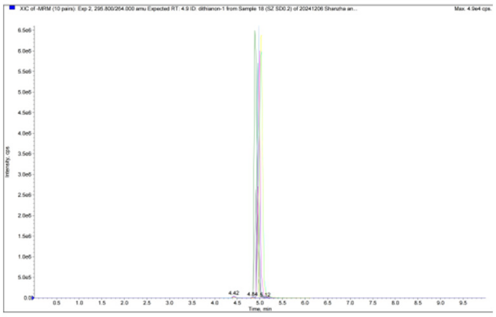

0.1 mg/kg

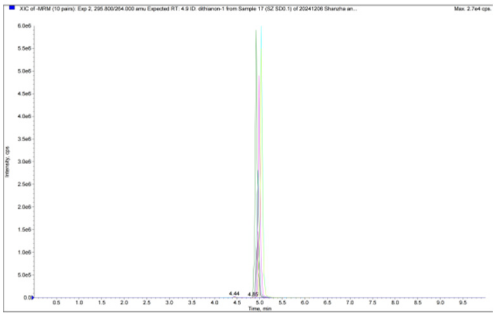

0.05 mg/kg

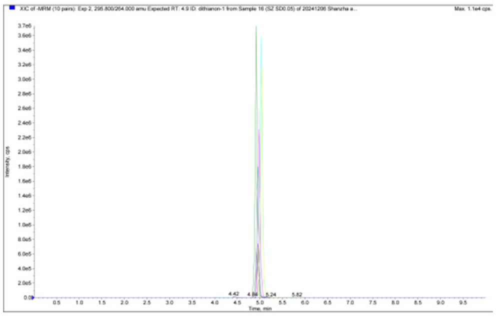

0.02 mg/kg

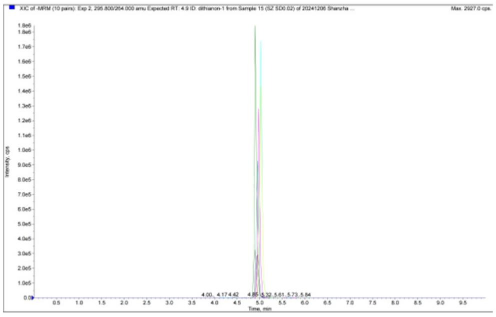

0.01 mg/kg

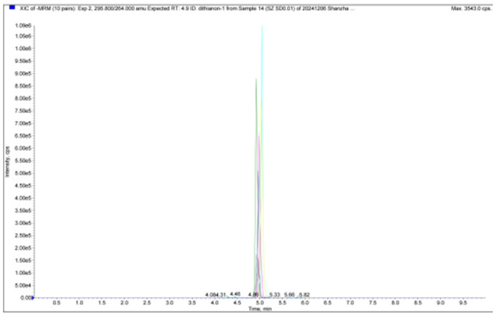

0.005 mg/kg

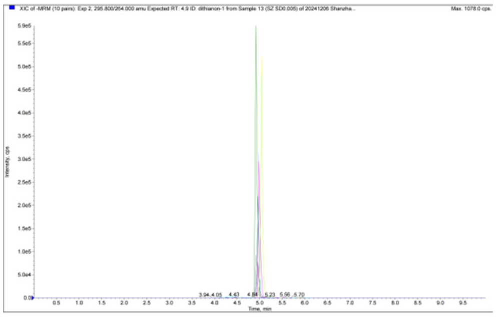

Figure.S4 Negative mode chromatogram during pesticide residue detection the mixed standard prepared with blank hawthorn sample as matrix.

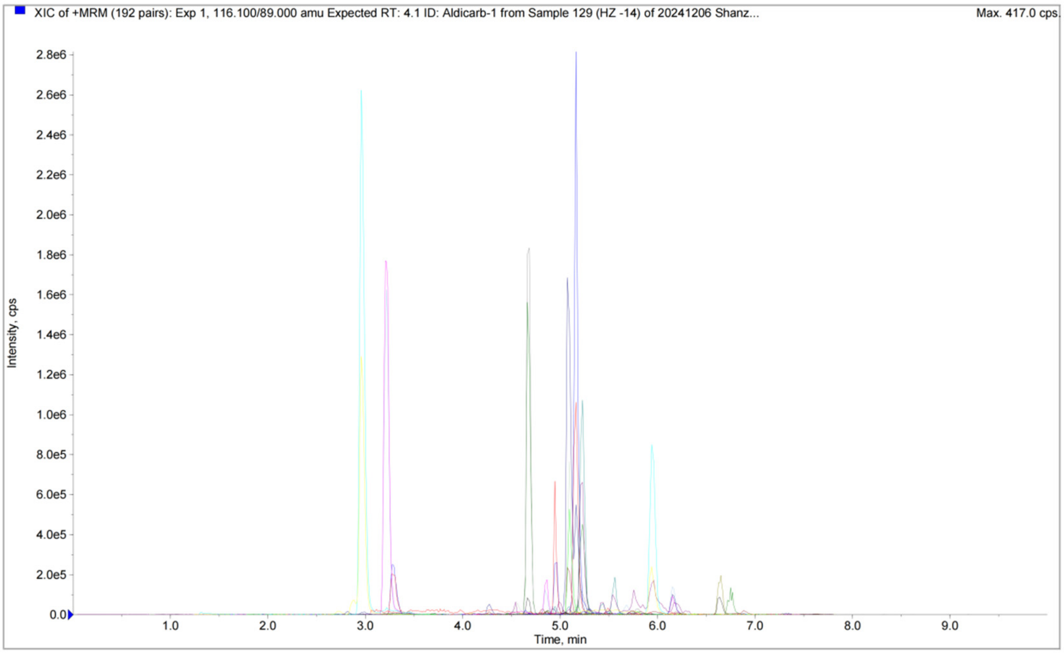

Figure.S5 Positive mode chromatogram in pesticide residue detection of fresh jujube samples.

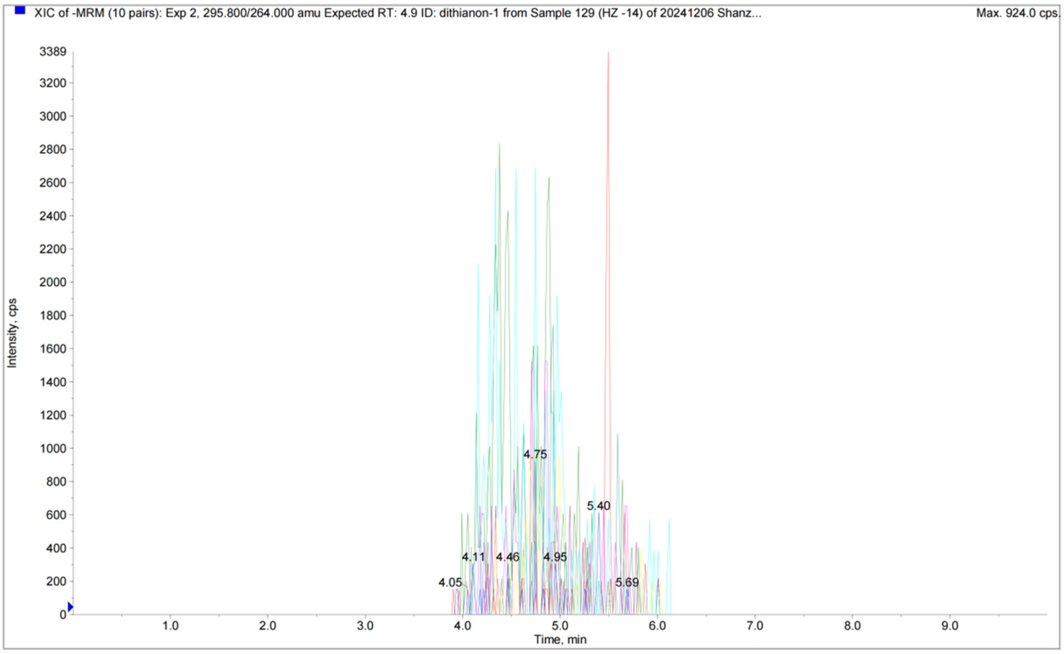

Figure.S6 Negative mode chromatogram in pesticide residue detection of fresh jujube samples.

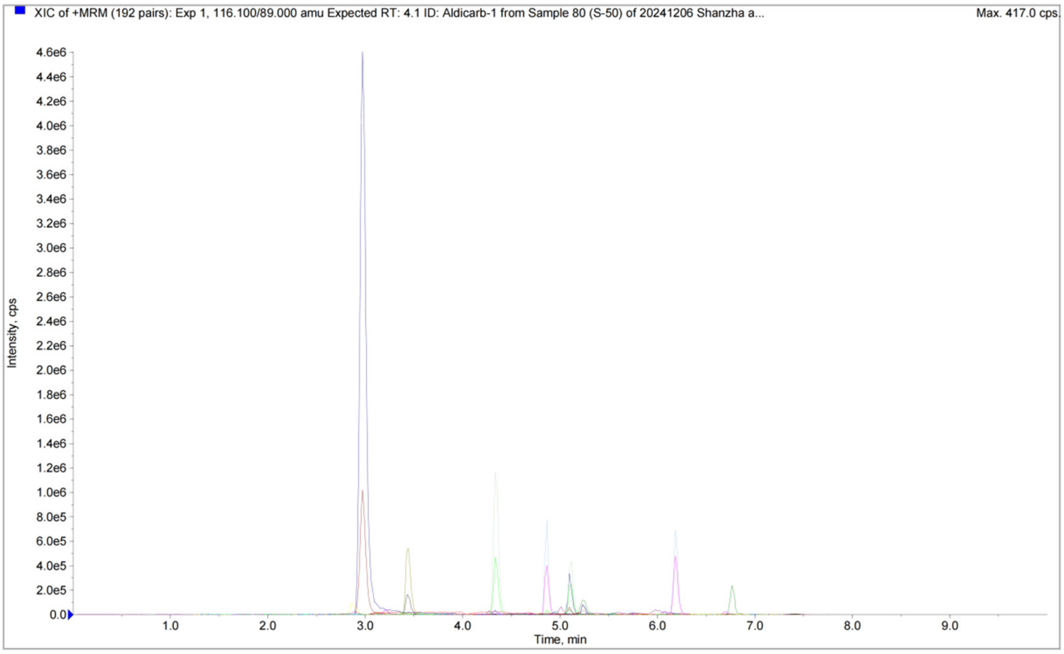

Figure.S7 Positive mode chromatogram in pesticide residue detection of hawthorn samples.

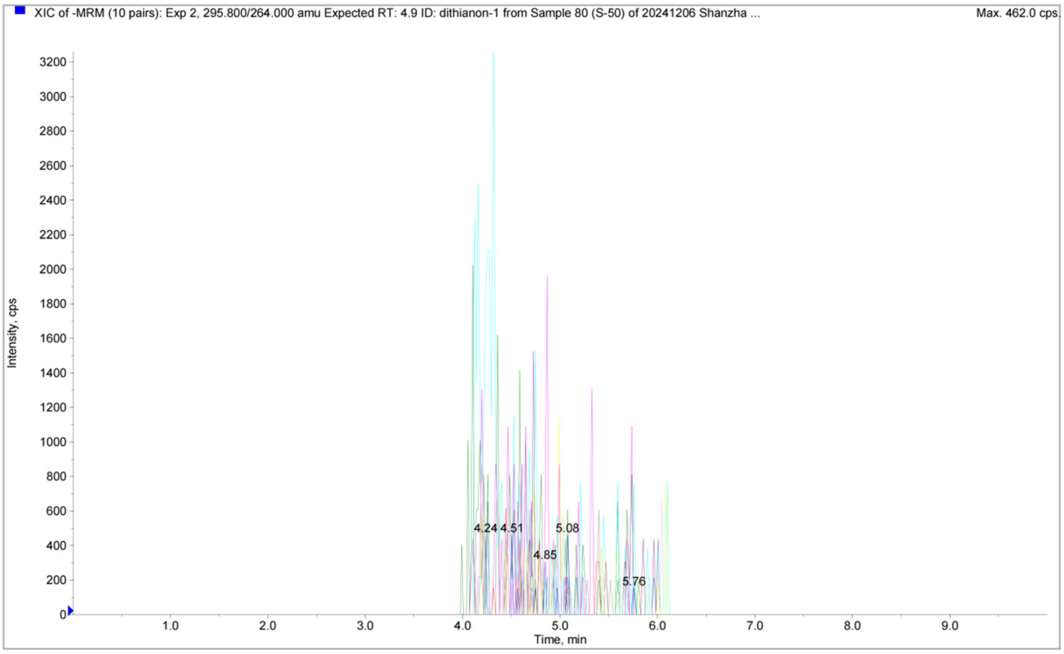

Figure.S8 Negative mode chromatogram in pesticide residue detection of hawthorn samples.
